# Factor Structure of the Experiences in Close Relationships—Relationship Structures Scale (ECR-RS) in Siblings of Children with Chronic Disorders

**DOI:** 10.3390/children11050560

**Published:** 2024-05-08

**Authors:** Krister W. Fjermestad, Stian Orm, Trude Fredriksen, Yngvild B. Haukeland, Torun M. Vatne

**Affiliations:** 1 Department of Psychology, University of Oslo, N-0373 Oslo, Norway; trude.fredriksen@sykehuset-innlandet.no (T.F.); tva@frambu.no (T.M.V.); 2 Frambu Resource Center for Rare Disorders, N-1404 Siggerud, Norway; 3 Innlandet Hospital Trust, N-2381 Brumunddal, Norway; stian.orm@inn.no; 4 Department of Psychology, Inland Norway University of Applied Sciences, N-2624 Lillehammer, Norway; 5 The Blue Cross Foundation, N-0179 Oslo, Norway; yngvild.haukeland@blakors.no

**Keywords:** siblings, parents, chronic disorders, psychometric evaluation, attachment, factor analysis

## Abstract

Our objective was to examine the factor structure of the Experiences in Close Relationships-Relationships Structures (ECR-RS), an attachment-theory based relationship measure, in at-risk sample comprising siblings of children with chronic disorders. Psychometric studies with general populations have demonstrated that the ECR-RS comprises two factors, representing anxiety and avoidance in close relationships. The sample comprised 103 siblings (M age = 11.5 years, SD = 2.2, range 8 to 16 years) of children with chronic disorders and their parents. The siblings completed a 9-item version of the Experiences in Close Relationships-Relationships Structures (ECR-RS) about their relations with mothers and fathers that was analyzed with confirmatory factor analysis. We examined construct validity using correlations between sibling social functioning, measured with the Strengths and Difficulties Questionnaire, and parent mental health, measured with the Hopkins Symptom Checklist-90-Revised. The ECR-RS comprised two factors, anxiety and avoidance, in line with previous studies. Both factors demonstrated significant overlap with sibling social functioning, but not with parental mental health. We conclude that the ECR-RS comprises two factors, anxiety and avoidance, that are related to siblings’ social functioning. The ECR-RS can be used as a psychometrically sound measure of relationship anxiety and avoidance in families of children with chronic disorders.

## 1. Introduction

Siblings of children with chronic disorders (herein; siblings of CwCD) are at increased risk of mental health problems (see [1,2] for the most recent reviews). Chronic disorders entail a heterogeneous set of illnesses or disabilities that are long-lasting and that enhances the child’s care needs over time. There is limited empirically based knowledge on which factors influence this risk, but family processes such as parental stress, care responsibilities, illness-related uncertainty, and poorer family communication are potential contributing factors (e.g., [1,3,4]. The mental health risk facing of siblings of CwCD indicate that these children may need interventions. However, as the risk facing siblings of CwCD is small (effect sizes *g* = 0.13 to 0.22, [1]), psychometrically sound measures to evaluate the individual need for intervention are needed.

Previous intervention trials for siblings of CwCD have been criticized for including sibling participants “just because” they are siblings of CwCD [5]. This practice is problematic as it implies unwarranted use of clinician and family time, wrong use of resources that could be used for families with larger needs, and also poor research quality, as including participants without a certain impact of the disorder may “wash out” intervention effects and lead to misinterpretation of outcome findings [2,6]. For these reasons, it is important to develop evidence-based measurements tailored for siblings of CwCD [5,7]. By making these available to service providers, siblings of CwCD who are at risk may be properly identified and individually tailored and effective interventions provided. An important step on the way towards establishing such an assessment base is to establish an evidence-base of psychometrically sound measures that can be used with siblings of CwCD.

Some recent attempts have been made to investigate the psychometric properties of measures used with siblings of CwCD. Orm and colleagues [8] examined the Negative Adjustment Scale, a measure developed for the sibling of CwCD population that includes sibling of CwCD-specific items such as “I worry about my brother’s/sister’s disease”. In a sample of 107 siblings, the authors found the scale to be a valid measure of adaptation to the sibling experience with some significant correlations with mental health in siblings of CwCD (*r* = 0.29 to 0.44) [8]. A disadvantage of this measure, despite its’ usefulness within the sibling of CwC population, is that it is not possible to use it to compare siblings of CwCD with children whose siblings have no disorder. Orm and colleagues [9] also examined a child communication measure, the Parent-Child Communication Scale (Conduct Problems Prevention Research Group, 1994), in a combined sibling of CwCD and community control sample, and found the original factor structure to be a good fit. The authors also found the Parent-Child Communication Scale to differentiate between siblings of CwCD and community controls with medium to large effect sizes, suggesting that the Parent-Child Communication Scale is one potential measure to identify siblings of CwCD at risk and in need of intervention [9]. Both these measures are particularly relevant for siblings. However, there are also other domains in need of psychometrically sound evidence.

A key challenge in families of children with chronic disorders is the strain that the disorder puts on interpersonal relations, including attachment patterns. In the pioneering work of Mary Ainsworth and colleagues in the 1970s, the two most fundamental insecure attachment patterns in children were labeled anxious and avoidant [10] (Although revisions and expansions of these attachment patterns have later been added, the fundamental anxiety and avoidance patterns have considerable empirical support as fundamental insecure patterns in interpersonal relations (e.g., [11,12]). These dimensions comprise the two factors of a widely used self-report measure for attachment patterns, i.e., the Experiences in Close Relationships—Relationship Structures Scale (ECR-RS; [13]), which is used in the current study.

Multiple versions of the ECR scales exist. The original 36-item ECR-RS [13] was designed as a measure of romantic attachment in adults. Later adaptions for attachment to parents from a child and adolescent perspective (12 and 36 item versions) have been developed [14,15], as well as shorter versions of the adult scale (9 items; [16]). In all versions, the ECR is meant to tap attachment-related anxiety (e.g., fear of abandonment or rejection) and avoidance (e.g., problems with closeness, self-reliance). The items are rated on a 7-point scale from “completely untrue” to “completely true”. In a review of attachment measures, Jewell and colleagues [17] rated the ECR-RS (36 and 9 item versions) as having no psychometric evidence beyond internal consistency, including lack of structural validity. This is a potential problem with the ECR-RS. Since this review [17], Sarling and colleagues [18] performed a confirmatory factor analysis on the 9-item ECR-RS with 806 adults and identified the proposed anxiety and avoidance dimensions. In 2014, Donbaek and Elkilit examined the factor structure of the 9-item ECR-RS used with adolescents aged 15–18 years and confirmed the proposed anxiety and avoidance dimensions [19]. They also found evidence for concurrent and discriminant validity in terms of overlap with other relationship measures and illegal drug use [19].

There have also been psychometric evaluations of child-adapted versions of the ECR. Brenning and colleagues [14] adapted the 36-item version of the ECR-RS to use with children and adolescents, simplifying formulations, removing double negatives, and adapting more to the child’s perspective on their relationship to their parents. Change examples include original item “I feel comfortable sharing my private thoughts and feelings with my partner” modified to “I find it easy to tell my mother/father what I think and how I feel” and original item “I don’t feel comfortable opening up to romantic partners” modified to “It’s not easy for me to tell my mother a lot about myself”. In a comprehensive two-stage study [14] the scale was adapted using clinician and child focus groups for feedback and statistically examined the factor structure of the 36-item revised child version with children and adolescents aged 8 to 14 years. They labelled the child-adapted version of the scale the ECR-RC, short for the Experiences in Close Relationships-Revised Child Version [14]. The Brenning team developed and validated a 12-item version of the ECR-RC in a psychometric study testing the ECR-RC with four different youth samples aged 12 to 18 years [15]. Skoczeń and colleagues [20] examined the factor structure of the full 36-item ECR-RC and the 12-item version (child versions). For both versions, the authors identified the avoidance and anxiety dimensions, as well as a possible third factor, security [20]. Marci and colleagues [16] later did a confirmatory factor analysis of the ECR-RC 12-item version and empirically identified the proposed anxiety and avoidance dimensions in a sample of Italian children. They also found evidence of concurrent and convergent validity in terms of overlap with children’s sense of social security and self-worth [16].

The majority of the psychometric evidence behind the different ECR-RS/RC versions is based on non-clinical populations. A few studies have been conducted with at-risk samples. Cimino and colleagues [21] found specific genetic influences on attachment quality based on the 12-item ECR-RC in children with disruptive mood disorder. Other teams have used the ECR-RS (i.e., the adult version) with youth samples, also demonstrating reliability and validity of the scale. In a sample of 98 children with persistent pain and their parents based on the 36-item ECR-RS, a study found that child attachment avoidance was related to higher parental strain and the use of fewer protective parenting behaviors [22]. A study of children with chronic pain based on the 36-item ECR-RS found that anxious attachment mediated the relationship between pain registration and quality of life [23].

The ECR-RS can be particularly valuable for the sibling of CwCD population for several reasons. First, the nature of chronic disorders may enhance children’s basic needs for safety and comfort from parents, such as seeking protection from danger (e.g., if the child with diagnosis has a violent tantrum) and comforted when in emotional pain (e.g., if the child with diagnosis has a medical emergency [24]). Second, less time with and attention from parents as due to their higher caregiving burden may be experienced by siblings of CwCD as differential treatment and complicate child-parent relations [25]. Third, parents, like siblings of CwCD, are at increased risk of stress and mental health problems, which may negatively affect close relationships [26]. Fourth, research indicates that interpersonal relations are affected, as siblings of CwCD have been found to try to cope with their challenges alone, and to not seek support from parents to the same extent as their peers [27]. Such avoidance is a central attachment mechanism. Fifth, siblings and parents represent the most important support network for children with chronic disorders [7]. Thus, these relations are likely to also affect the health of the child with disorders, so finding ways of assessing relational risks is of use to the field. Finally, there is a particular practical advantage of using the 9-item version of the ECR-RS, as we did in the current study, as it is short and thus reduces the burden on informants.

To our knowledge, few studies have assessed attachment quality in siblings of CwCD. A study compared 56 siblings of children with rare disorders to 44 community controls and found that siblings reported more avoidance in the relationship with their mothers (effect size difference *d* = 0.70) and more avoidance (*d* = 0.91) and anxiety (*d* = 0.47) in the relationship with their fathers, using the 9-item ECR-RS [28]. Also using the 9-item ECR-RS, a later study found a composite score of attachment avoidance and anxiety in the relationship with mothers and fathers, respectively, to be the only significant predictor across all informants (self, mother, father) of siblings’ mental health [28]. Parent-child communication, siblings’ adjustment to the disorder, and parental mental health were inconsistent predictors in comparison. Thus, these two studies suggest that attachment is an important domain in sibling research [27,28].

Despite these promising findings, the field lacks a psychometric evaluation of the 9-item ECR-RS used with an at-risk sibling of CwCD sample. Both factor structure and validity may be different for younger children and at-risk samples. Furthermore, in previous studies using the ECR-RS with siblings of CwCD, one study used the proposed two-factor solution and reported acceptable internal consistency (α = 0.75 to 0.82; [28]), whereas the other study collapsed the avoidance and anxiety dimensions into one composite score and reported acceptable internal consistency (α = 0.76 to 0.78; [29]).

The aim of the current study was to examine the factor structure of the 9-item ECR-RS in a sample comprising siblings of CwCD. To examine concurrent validity, we included a measure of the siblings of CwCD’s prosocial behavior and peer problems [30]. These dimensions are relevant for the anxiety dimension of the ECR-RS, which includes social worries, and for avoidance, which includes self-reliance. Furthermore, secure attachment (i.e., low avoidance and anxiety) has been found to be related to a more prosocial orientation of peer relationships in previous research [31]. We also included parent mental health, which is well-documented to influence parent-child relations [32]. We expected that the anxiety and avoidance dimensions would be replicated in the current sample. We expected that better child-parent relations would be associated with higher prosocial behavior, less peer problems, and better parental mental health in sibling of CwCD.

## 2. Methods

### 2.1. Participants

The sample comprised 103 siblings of CwCD and their parents. The siblings of CwCD were aged 8 to 16 years (M = 11.5 years; SD = 2.1; 54% girls, 46% boys). The disorders were rare disorders (52%), autism spectrum disorder (25%), congenital heart diseases (12%), Down syndrome (7%), or cerebral palsy (4%). Mothers’ mean age was 41 years (SD = 4.9). Fathers’ mean age was 44 years (SD = 5.5).

### 2.2. Setting and Procedures

Siblings of CwCD and parents were recruited from two specialist centers (for rare disorders and autism, respectively) and user associations for autism, cerebral palsy, congenital heart disease, or Down syndrome. Participants were invited to take part in an intervention study that took place throughout Norway. The inclusion criteria were age 8 to 16 years and having a brother or sister with a rare disorder, autism spectrum disorder, congenital heart disease, Down syndrome, or cerebral palsy. One parent needed to be able to attend the intervention. Further details on the trial have been reported elsewhere [33]. The current study only focuses on the baseline data.

### 2.3. Ethical Considerations

Written informed consent was obtained from all parents, on behalf of themselves and the children. The families were informed that participation was voluntary. No financial incentives were offered. The study was approved by and conducted in accordance with the Regional Committees for Medical and Health Research Ethics.

### 2.4. Measures

*The Experiences in Close Relationships—Relationship Structures Questionnaire* (ECR-RS) [13] was used to measure the quality of siblings of CwCD’s relationship with fathers and mothers, i.e., reported for each parent. The 9-item ECR-RS is a self-report instrument designed to assess attachment patterns in a variety of close relationships. Siblings of CwCD rated their relationship with parents on a 7-point scale from 1 (correct) to 7 (incorrect) (e.g., “It is easy for me to trust my mother/father”). The questionnaire is divided into two subscales based on the two fundamental dimensions underlying attachment patterns: anxiety (six items) and avoidance (three items) [13]. Satisfactory reliability has been reported for the ECR-RS (α = 0.88 to 0.92) [34]. The ECR-RS was developed based on a large population study with >20,000 adults recruited online [34], but has also been used with non-clinical school-based child populations [16]. The current study is, to the best of our knowledge, the first psychometric evalutation of the ECR-RS with Norwegian children.

*The Strengths and Difficulties Questionnaire* (SDQ) [35] was used as a measure of siblings’ peer problems and prosocial behavior. We used two subscales; (1) peer problems (5 items, e.g., often fights with other children or bullies them), and (2) prosocial behavior (5 items, e.g., shares readily with other children [36]. Items are rated on a Likert-scale from not true (0) to certainly true (2). The internalizing and externalizing subscales concern mental health problems whereas the prosocial behavior subscale represents strengths. The SDQ has demonstrated adequate psychometric properties [36]. In the current study, siblings and parents completed the SDQ. Internal consistency analyses showed adequate reliability across subscales and informants (M α = 0.66). The official Norwegian translation of the SDQ (sdqinfo.org) was used.

*The Hopkins Symptom Checklist-90-Revised* (SCL-90-R) [37] was used as a measure of parent mental health. The SCL-90-R is a 90-item self-report questionnaire where items describing various mental health symptoms experienced during the last seven days are rated 0 (not at all) to 4 (very much). We used the SCL-90-R global severity index in the current study. The SCL-90-R has documented psychometric properties and is widely used in adult mental health services [38]. In the current sample, internal consistency (Cronbach’s α) was good for mothers (α = 0.96) and fathers (α = 0.97). The official Norwegian translation of the SCL-90 was used [38].

### 2.5. Data Analytic Plan

We compared a one-factor versus a two-factor model of the ECR-RS with confirmatory factor analyses (CFA) for report on mother and father, respectively. We used the statistical software JASP (Version 0.17.3) [39] and diagonally weighted least square (DWLS) estimator. Following fit indices were used to determine goodness of fit: non-significant chi-square test, root mean square error approximation (RMSEA) < 0.08 with *p*-close > 0.05, comparative fit index (CFI) and Tucker-Lewis Index (TLI) > 0.95, and standardized root mean square residual (SRMR) < 0.10 [40]. To compare models, we also used the Expected Cross Validation Index (ECVI) with a lower value indicating a better model. The amount of missing data on ECR items was low (≤4.9%) and Missing Completely At Random (MCAR) according to Littles test (*p* = 0.614; [41]). To retain power, missing values were accommodated using the Expectation-Maximization (EM) procedure to give maximum likelihood imputation of missing values in SPSS version 27 [42]. We also ran the models without imputing missing values and findings remained the same. To examine the overlap between the ECR-RS and prosocial behavior and peer problems (SDQ), and parental mental health (SCL-90-R), we computed Pearson’s *r* in SPSS version 27. Pearson’s *r* of 0.10, 0.30, and 0.50 were considered a small, medium, and large effect size, respectively [43].

## 3. Results

### 3.1. Confirmatory Analyses

**Figure 1:**
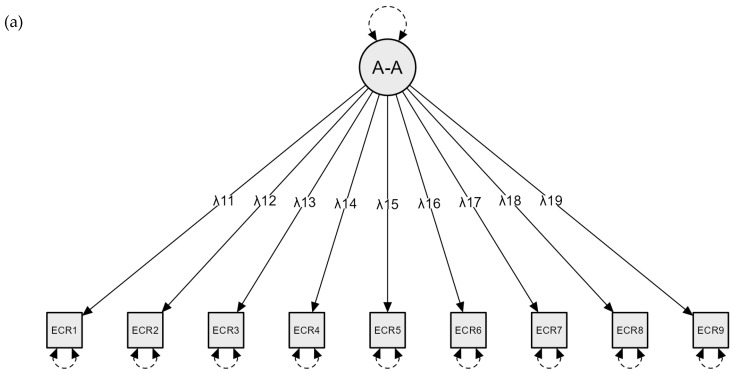

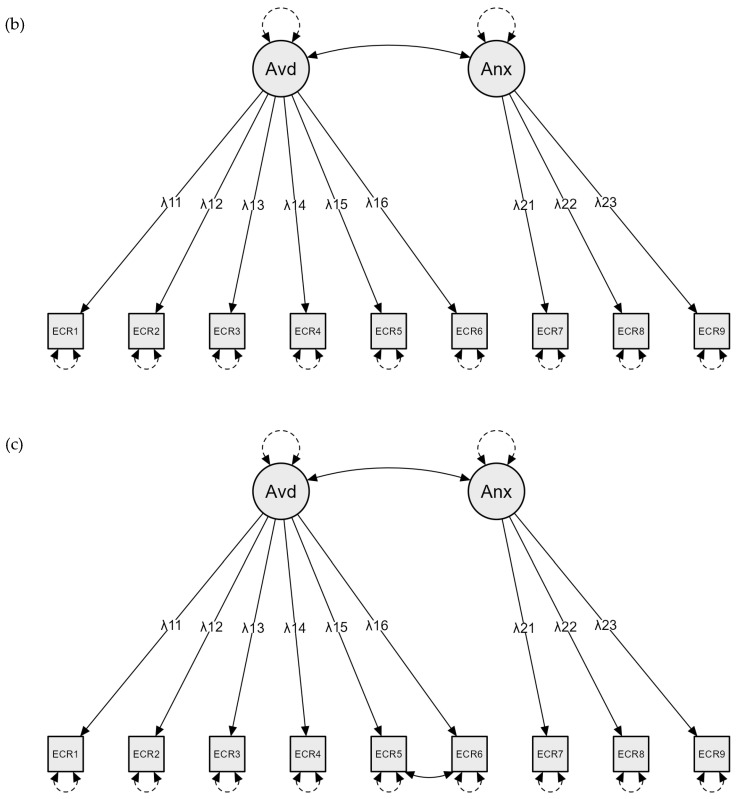
Conceptual Figures of the Three Different Factor Models. Note. (**a**) The one-factor model where relationship avoidance and anxiousness were collapsed into a unitary factor. (**b**) The two-factor model of the adult version of the ECR-RS, with one factor for avoidance and one for anxiousness. (**c**) The modified two-factor model, where the solid, curved line with bidirectional arrows between item 5 (ECR5) and 6 (ECR6) illustrates the residual covariance between the two items that were included in the model.

See Figure 1 for illustrations of the different factor solutions. The one-factor solution showed poor fit for report about mother and marginal fit for report about father on the ECR-RS (see Table 1). For the original two-factor solution, report about father showed good model fit whereas report about mother still showed unsatisfactory model fit. Inspection of modification indices showed high residual covariance (i.e., correlated measurement error [CME]) between item 5 and 6 on both report about father (modification index [MI] = 10.365) and report about mother (MI = 22.310). When inspecting these two items this makes sense, as they are conceptually similar (i.e., item 5: “I don’t feel comfortable opening up to my mother/father”; item 6: “I prefer not to show my mother/father how I feel deep down”). Thus, we included the CME into the model to see whether this would improve model fit. The modified two-factor model with CME showed excellent model fit with lower ECVI than previous models for both report about father and report about mother (Table 1), suggesting that this model fitted the data best. All factor loadings were significant for report about father (all *p* < 0.001) and mother (all *p* ≤ 0.008).

Reliability analyses of internal consistency showed good internal consistency for ECR-RS anxiety about mothers (ω = 80, 95% CI [0.66, 0.90], α = 0.79, 95% CI [0.64, 0.89]), about fathers (ω = 84, 95% CI [0.72, 0.92], α = 0.80, 95% CI [0.62, 0.90]), and for ECR-RS avoidance about mothers (ω = 73, 95% CI [0.61, 0.82], α = 0.75, 95% CI [0.64, 0.82]), and about fathers ω = 78, 95% CI [0.66, 0.85], α = 0.79, 95% CI [0.67, 0.85]). See Table 2 for the factor loadings for the mother and father versions, respectively.

### 3.2. Convergent Validity

Both attachment factors, rated about both parents, evidenced significant overlap with child-rated peer problems. All factors except anxiety in relation to father were significantly correlated with child-rated prosocial behavior. Both anxiety dimensions (father and mother) were significantly related to mother-rated peer problems. Both avoidance dimensions (father and mother) were significantly related to father-rated peer problems and prosocial behavior. Maternal avoidance was significantly related to mother-rated prosocial behavior. There were no significant associations between the attachment dimensions and parent mental health. See Table 3.

## 4. Discussion

We identified that the ECR-RS evidenced two factors, anxiety and avoidance, in a sample of siblings of CwCD who rated the relationship quality to their mothers and fathers. Our findings correspond to the handful of previous studies that have examined the ECR-RS. Donbaek and Elkilit [19] examined the factor structure of the 9-item ECR-RS with adolescents and confirmed the anxiety and avoidance dimensions. They also found evidence for concurrent and discriminant validity in terms of overlap with other relationship measures and illegal drug use [19]. Another team [20] examined the factor structure of the 36-item version of the ECR-RS and identified the avoidance and anxiety dimensions. Yet another group [18] performed a confirmatory factor analysis on the 9-item ECR-RS with adults and identified the anxiety and avoidance dimensions. Finally, one study [16] did a confirmatory factor analysis of the ECR-RC 12-item child-adapted version and identified the anxiety and avoidance dimensions in Italian children. The authors also found evidence of concurrent and convergent validity in terms of overlap with child social security and self-worth [16].

The strengths of the current study include the focus on an at-risk group with a sample size that is sufficient for examining the factor structure of a 9-item scale. There are also limitations. The sample represents a wide range of disorders, so albeit the representativeness is broad, it cannot be determined if relationship structures vary by disorder. For example, disorders involving conduct problems and thus family disruption may affect family relations differently from somatic disorders and physical disabilities. A central issue concerns the use of the original ERC-RC, developed for adults, when a well-validated child version exists [14,15]. However, it is important to note that there are very small differences in formulations between the 9-item ECR-RS used in the current study and the 12-item ECR-RC. Of particular relevance to the current study, the items were also translated to Norwegian for use with children and adolescents, and the nuance differences between the original adult version, the adapted child version, and the Norwegian translations can be considered minimal. See Table 4 for overview. Lastly, some concerns have been raised around the modelling of CME in CFA analyses, as this may contribute to fitting a model to the idiosyncrasies of a given sample at the expense of moving away from the true underlying population model [44]. Thus, future cross-validation with other sibling samples is important. However, in a general-population sample of Swedish adults, a study also found that model fit improved substantially by including the CME between item 5 and 6, suggesting that there may be a true methods effect (i.e., similar wording, conceptual overlap) between the two items [18].

The main implication of the current study is that the 9-item version of the ECR-RS can be reliably used with siblings of CwCD. This is important, since albeit the needs of siblings of CwCD are being increasingly recognized internationally, there are no gold standards for assessing their intervention needs [2]. Given that family relations may be strained due to the challenges a chronic disorder represents, a well-validated relationship measure can be a useful part of an assessment [3]. From a practice perspective, the shortness of the 9-item ECR-RS is a considerable advantage, in particular if it is used in combination with other measures. We conclude that the 9-item ECR-RS is a structurally sound measure for the sibling population. As such, the measure can be considered part of the increased portfolio of measures available when considering the risk facing siblings and the assessment of their need for intervention. This should be considered alongside other important domains of functioning such as mental health and quality of life.

## Figures and Tables

**Table 1:** Fit Indices for the Different Factor Models of the ECR-RS.

			χ^2^	*p*	RMSEA	*p*	CFI	TLI	SRMR	ECVI
Model										
	Report about father									
		One-factor	30.768	0.281	0.08	0.606	0.98	0.97	0.14	0.655
		Two-factor	23.618	0.598	0.00	0.850	1.00	1.02	0.10	0.604
		Two-factor modified	13.040	0.976	0.00	0.996	1.00	1.10	0.09	0.520
	Report about mother									
		One-factor	61.641	<0.001	0.11	0.005	0.77	0.69	0.17	0.957
		Two-factor	41.428	0.028	0.08	0.158	0.90	0.86	0.11	0.779
		Two-factor modified	18.410	0.825	0.00	0.952	1.00	1.06	0.08	0.573

Note. RMSEA = Root Mean Square Error of Proximation. CFI = Comparative Fit Index. TLI = Tucker Lewis Index. SRMR = Standardized Root Mean Squared Error. ECVI = Expected Cross Validation Index.

**Table 2:** Factor Loadings Two-Factor Models Modified for Mother and Father.

						95% Confidence Interval	
Factor	Item	Estimate	SE	z-Value	*p*	Lower	Upper
Factor 1	1. It helps to turn to my mother in times of need *	1.040	0.174	5.968	<0.001	0.698	1.381
	2. I usually discuss my problems and concerns with my mother *	1.221	0.197	6.210	<0.001	0.835	1.606
	3. I talk things over with my mother *	0.838	0.141	5.925	<0.001	0.561	1.115
	4. I find it easy to depend on my mother *	1.042	0.182	5.720	<0.001	0.685	1.399
	5. I don’t feel comfortable opening up to my mother	−0.345	0.130	−2.646	0.008	−0.601	−0.089
	6. I prefer not to show my mother how I feel deep down	−0.571	0.135	−4.228	<0.001	−0.836	−0.306
Factor 2	7. I often worry that my mother doesn’t really care for me	1.286	0.266	4.832	<0.001	0.764	1.807
	8. I’m afraid that my mother may abandon me	1.218	0.279	4.365	<0.001	0.671	1.765
	9. I worry that my mother won’t care about me as much as I care about her	0.934	0.226	4.124	<0.001	0.490	1.377
Factor 1	1. It helps to turn to my father in times of need *	0.985	0.146	6.764	<0.001	0.700	1.271
	2. I usually discuss my problems and concerns with my father *	1.355	0.176	7.698	<0.001	1.010	1.699
	3. I talk things over with my father *	1.249	0.169	7.384	<0.001	0.917	1.580
	4. I find it easy to depend on my father *	0.762	0.129	5.909	<0.001	0.509	1.015
	5. I don’t feel comfortable opening up to my father	−0.498	0.124	−4.013	<0.001	−0.742	−0.255
	6. I prefer not to show my father how I feel deep down	−0.813	0.139	−5.844	<0.001	−1.085	−0.540
Factor 2	7. I often worry that my father doesn’t really care for me	1.101	0.215	5.135	<0.001	0.681	1.522
	8. I’m afraid that my father may abandon me	0.993	0.219	4.532	<0.001	0.564	1.422
	9. I worry that my father won’t care about me as much as I care about her	0.983	0.236	4.168	<0.001	0.521	1.446

Note. Norwegian translation of the items used in the current study. * Item reverse coded. SE = Standard Error.

**Table 3:** Correlations Between the ECR-RS Factors and Other Measures (Convergent Validity).

	Peer Prob.-C	Prosocial-C	Peer Prob.-M	Prosocial-M	Peer Prob.-F	Prosocial-F	Ment. Health-M	Ment. Health-F
Avoidance F	0.28 **	−0.33 ***	0.16	−0.20	0.29 **	−0.32 **	0.03	0.19
Anxiety F	0.23 *	0.03	0.23 *	−0.08	0.09	−0.17	0.08	−0.11
Avoidance M	0.22 *	−0.26 *	0.16	−0.20 *	0.23 *	−0.40 ***	0.04	0.19
Anxiety M	0.34 ***	0.02	0.25 *	−0.11	0.07	−0.12	0.05	−0.11

Note. F = Father. M = Mother. C = Child. Prob. = problems. Ment. = mental. * Correlation is significant at the *p* < 0.05 level. ** Correlation is significant at the *p* < 0.001 level. *** Correlation is significant at the *p* < 0.0001 level.

**Table 4:** Original Adult versus Child-Adapted Item Formulations.

9-Item Version Used in the Current Study	Corresponding or Similar Item in the 12-Item Child-Version
It helps to turn to my mother in times of need	When I feel bad, it helps to talk to my mother
I usually discuss my problems and concerns with my mother	I usually talk to my mother about my problems and worries
I talk things over with my mother	I tell my mother nearly everything
I find it easy to depend on my mother	I prefer not to get too close to my mother
I don’t feel comfortable opening up to my mother	It’s not easy for me to tell my mother a lot about myself
I prefer not to show my mother/father how I feel deep down	I don’t like telling my mother how I feel deep down inside
I often worry that my mother doesn’t really care for me	I’m worried that my mother doesn’t really love me
I’m afraid that my mother may abandon me	I’m worried that my mother might want to leave me
I worry that my mother won’t care about me as much as I care about her	I’m worried that my mother doesn’t love me as much as I love her

Note. Similar version where “father” is used for mother exist. Child-adapted version by [15].

## Data Availability

The data presented in this study are available on request from the first author due to the regulations set by the relevant data protection agency. Only anonymized data can be shared.
